# Dietary Fat Intake and Indices of Blood Profiles in High-Performance Athletes: An Exploratory Study Focusing on Platelet Variables

**DOI:** 10.3390/nu17213418

**Published:** 2025-10-30

**Authors:** Marius Baranauskas, Ingrida Kupčiūnaitė, Jurgita Lieponienė, Rimantas Stukas

**Affiliations:** 1 Faculty of Biomedical Sciences, State Higher Education Institution Panevėžys College, 35200 Panevėžys, Lithuania; ingrida.kupciunaite@panko.lt (I.K.); jurgita.lieponiene@panko.lt (J.L.); 2 Department of Public Health, Institute of Health Sciences, Faculty of Medicine, Vilnius University, 01513 Vilnius, Lithuania; rimantas.stukas@mf.vu.lt

**Keywords:** blood profiles, nutritional status, omega-3 fatty acids, platelet variables, public health

## Abstract

**Background**/**Objectives**: There is a sudden and noticeably increasing focus on naturally found antiplatelet inhibitors that humans can use habitually. Given that athletes receive annual training with periods of recovery that are not always suitably adapted to the workload, this study aimed to explore the association between dietary fat intakes and the indices of blood profiles, concentrating on platelet variables in a sample of high-performance athletes. **Methods**: The sample encompassed 19.8 ± 2.2-year-old Lithuanian high-performance athletes (*n* = 82). The assessment of the nutritional profile of study participants was performed using a 3-day food record approach. In laboratory settings, the hematology profile of athletes was assessed via the Nihon Khoden automated hematology analyzer. **Results**: The recorded mean consumption of energy, carbohydrates, protein, and fat in elite athletes was 49 kcal/kg/day, 5.4 g/kg/day, 1.6 g/kg/day, and 40.3% of energy intake (EI), respectively. The study highlighted the excessive consumption of saturated fatty acids (FA) (13.4–14.3% of EI) and dietary cholesterol (698–982 mg/day). Also, considering that the ideal human omega-6 to omega-3 FA ratio is commonly deemed to be between 1:1 and 4:1, an athlete’s ‘Western diet’ was heavily skewed with a ratio fluctuating from 18.9:1 to 19:4 in favor of omega-6 FA. Furthermore, the study found that the outcomes related to slightly higher levels of blood platelet counts and plateletcrit, however, being within normal limits, were associated with a higher intake of omega-6 FA (adjusted odds ratio (AOR) 9.5, 95% confidence interval (CI) 1.2; 9.9, *p* = 0.029). A higher platelet-to-hemoglobin ratio as a novel indirect blood-based biomarker pronouncing the potential inflammatory processes in the body revealed the reverse relationship of higher intake levels of dietary omega-3 FA (AOR 6.7, 95% CI 1.3; 12.2, *p* = 0.029), omega-6 FA (AOR 6.2, 95% CI 2.7; 11.5, *p* = 0.009), and saturated FA (AOR 8.5, 95% CI 1.5; 9.1, *p* = 0.020) among elite athletes. **Conclusions**: The prospect of personalized nutrition targeted at the professional athletes’ segment may provide an innovative opportunity to increase athletes’ capacity to manage the platelet function via diet while stressing the importance of further empirical experimental research in this dynamic and vital biomedical field.

## 1. Introduction

It has been well established that dietary habits play a potential role in influencing the indices of blood profiles, including the platelet function, a fundamental component in the blood coagulation cascade [1]. It should be emphasized that thrombocytes, or platelets, are the smallest cell elements in the human blood [2] that play a significant role in supporting the equilibrium of hemostasis [3,4,5,6]. Derived from the bone marrow cells that mature into platelets [7], these non-nucleated cell fragments circulate in the blood circulation as precursors capable of responding to vascular damage [8,9]. Upon detecting an injury, platelets join the lesion area, aggregate with each other, and start a cascade of reactions. This results in a hemostatic stopper that effectively prevents further bleeding [10,11]. In addition, platelets are engaged in many other physiological processes, including angiogenesis, immunity, and inflammation, as significant agents under numerous physiological and pathological circumstances [12].

More specifically, a decreased activation response of thrombocytes may result in excessive bleeding, whereas platelet hyperactivity may lead to thrombotic complications. Additionally, platelet hyperactivity may be associated with various processes such as cancer metastasis, angiogenesis, the alteration of liver tissue, atherosclerosis, and an inflammatory and immune response [13,14,15,16], as well as many other platelet-mediated diseases, namely, cardiovascular disease (CVD) [17,18,19], Alzheimer’s disease, renal diseases, arterial and venous thrombosis, and microorganism infections [14,16,20,21,22]. However, the explanatory mechanisms have not been well studied. Thus, the assurance of regular thrombocyte aggregation is essential in averting platelet-related diseases in the conventional human population [23,24,25,26,27].

Against this background, the exception should also be made to the athletes’ population, where the activity of the bodily endothelial cells in athletes is rigorously related to the function of thrombocytes. In an abnormal state, the endothelial dysfunction can magnify the endothelial synergy with platelets [28]. Therefore, regular physical activity promotes blood circulation and shear stress, resulting in the increased bioavailability of nitric oxide (NO), which, in turn, is accompanied by its beneficial effects on endothelial function [29]. As a consequence, the occurrence of more intense blood flow and fluctuations in hemodynamics triggered by high physical loads may lead to both chronic and acute alterations in vascular activity.

High-intensity training, especially in some sports, can give rise to immoderate platelet aggregation and increase the risk of blood clot events. Science has long considered marathons and triathlons as sports related to a certain risk for thrombotic events [30]. Acute strength exercises may contribute to platelet activation, too [31]. Although the promotion of endothelial function has been demonstrated by an increased NO availability, the exact pathways underlying this phenomenon remain unresolved. However, these exercise-related outcomes associated with the platelet function are clearly similar to a well-established relationship between workouts and the risk for adverse cardiovascular events, where strenuous physical loads temporarily increase the risk for cardiac infarction while a healthy lifestyle dramatically diminishes deaths from heart and circulatory disorders [31].

As a result of the above-mentioned, there is a sudden and noticeable increase in focus on naturally found antiplatelet inhibitors that humans can use habitually. Research has identified a large number of dietary antiplatelet nutrients that may decrease the hyperactivity of platelets without any adverse effects [32,33,34,35,36]. For example, in terms of human nutrition, dietary fatty acids (FAs) can either trigger or inhibit platelet aggregation. Omega-3 FAs, mainly found in fish oil, has been shown to wield antiplatelet effects. Dietary omega-3 long-chain polyunsaturated FAs regulate platelet activity through the thromboxane B3 (TxB3) synthesis, the alteration of platelet membranes, and the acceleration of NO synthesis [14,32,37]. Therefore, these useful omega-3 FAs incorporate into platelet cell membranes, modifying their functionality and fluidity [38]. On the other hand, pro-inflammatory omega-6 FA, mainly found in vegetable oils as well as in seeds, eggs, nuts, poultry, and whole grains, can stimulate platelet aggregation.

Generally, it is important to highlight that the positive adaptive response, along with phenotypic alterations, depends upon increased performance, which in turn relies on the appropriate recovery time between each training course, too. High-performance athletes receive annual training and participate in competitions with periods of recovery that are not always suitably adapted to the workload. Thus, in view of the interrelation between the training courses, the nutritional status, and the variations in essential blood parameters, it is important to detect all the pathways in which the adaptation of the athlete’s body to physical loads can be optimized. In this connection, although the actual dietary habits play a vital role in determining the platelet function, a core element in the body’s clotting process [1], the outstanding relationship between the platelet function and the macronutrient intake amongst high-performance athletes remains largely unanalyzed and unexplained at present. For sequential arrangements, to fill the existing specific scientific gaps, this study aimed to explore the association between dietary fat intake and the indices of blood profiles, concentrating on platelet variables in a sample of high-performance athletes.

## 2. Materials and Methods

### 2.1. Study Design, Population, and Data Collection

A single cross-sectional study in design was conducted at the Lithuanian Sports Centre (LSC) between April and August in 2021–2022. A representative sample size (*n* = 75–176) with a constructed confidence interval of a 95% confidence level and a marginal error of 5–10% was obtained from the eligible population (*N* = 322) of elite athletes when applying the web-based online freeware OpenEpi version 3.01 [39].

Out of the eligible population, the following 176 high-performance athletes were included in the study using a probability sampling method, taking into account the estimated representative sample size and the inclusion criteria as follows: (1) athletes who trained for the Olympics; (2) study participants who represented a country as national or international athletes; (3) athletes who engaged in a high-frequency, six-day-per-week training course; (4) athletes in the preparation phase.

**Figure 1:**
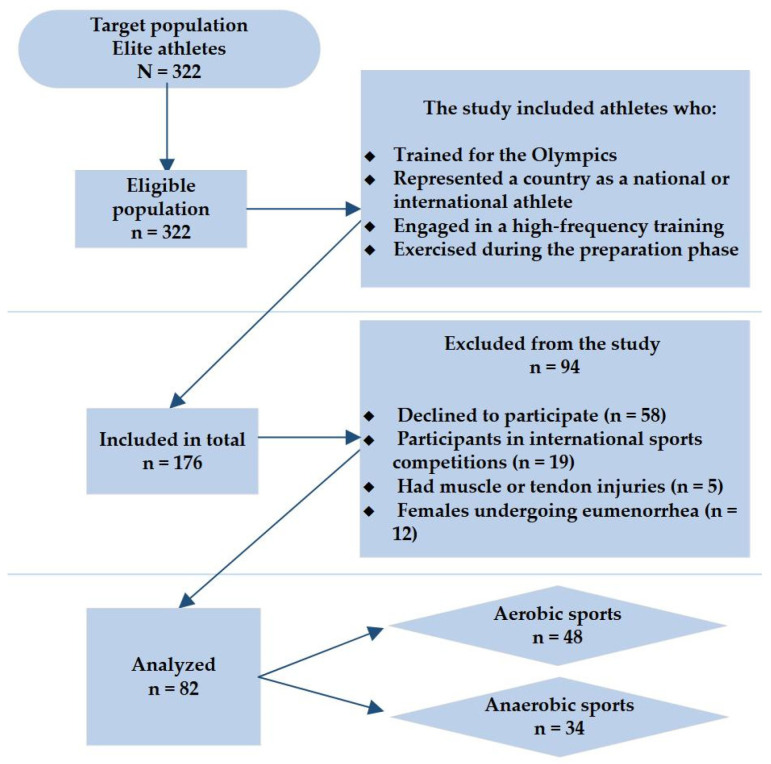
Enrollment flowchart for high-performance athletes.

The athletes were collected from the authorized list established by the National Olympic Committee of Lithuania (LTOK), the authorized body responsible for the Olympic movement in Lithuania [40]. The exclusion criteria were personalized as follows: (1) athletes who were vigorously competing in their respective sports during the study period (*n* = 19); (2) athletes who have reported muscle, tendon, or ligament injuries (e.g., hamstring strains, Achilles tendon issues, ankle sprains) (*n* = 5); (3) female athletes throughout their regular menstrual cycle (*n* = 12); (4) specific athletes who abstained from participating in the cross-sectional study (*n* = 58). Hence, healthy and non-medicated athletes were included in the study. A more detailed scheme for the enrollment process is displayed in Figure 1. Finally, based on a priori metabolic pathways (in terms of aerobic and anaerobic) in the body [41], the professional athletes (*n* = 82) were divided into two groups as follows: aerobic athletes ((*n* = 48: swimmers (*n* = 13), rowers (*n* = 2), modern pentathlon players (*n* = 5), road cyclists (*n* = 7), skiers (*n* = 8), biathletes (*n* = 7)) and boxers (*n* = 6)) and anaerobic athletes (*n* = 34: Greco-Roman wrestlers (*n* = 8), judokas (*n* = 8), and basketball players (*n* = 18)).

### 2.2. Measurements

#### 2.2.1. Dietary and Body Weight Assessment

The assessment of the athletes’ nutritional profile was performed using a 3-day food record approach where high-performance athletes tracked all food, dishes, and beverages over three consecutive days, aiming to evaluate a typical dietary intake and the levels of macronutrients consumed [42]. The specific food items, including portion sizes (using weight or measures), cooking approaches, food brands, and meal timing, consumed by athletes were recorded by a sports nutritionist through direct interviews [43], while the special Atlas of Foodstuffs and Dishes [44] for research purposes was also used. The energy intake (EI) and dietary macronutrient (carbohydrates, protein, lipids, saturated fatty acids (FA), cholesterol, monounsaturated FA, polyunsaturated FA, omega-6 FA, and omega-3 FA) intakes were assessed via the Nutrition Baseline software (NutriSurvey, the English translation of a commercial German software (EBISpro version 3.02), SEAMEO-TROPMED RCCN-University of Indonesia) [45]. The typical food dataset of NutriSurvey was manually augmented by the nutritional values of foodstuffs extracted from the Lithuanian chemical composition tables [46].

The intakes of macronutrients by the high-performance athletes we studied were evaluated in agreement with the Recommended Dietary Allowances (RDAs) established by the scientific recommendations [47,48,49,50,51]. Therefore, the recommended carbohydrate intake for athletes must be equal to 7–10 g/kg of body weight (BW), and protein intake must comprise 1.2–2.0 g/kg of BW. The total dietary fat percentage of EI should fluctuate from 25 to 35%, the saturated FA percentage must be <10% of EI, the polyunsaturated FA percentage should range from 6 to 10% of EI, the omega-6 FA percentage must vary between 5 and 8% of EI, omega-3 FA percentage should be ≥2% of EI, and the omega-6 FA to omega-3 FA ratio should be in a healthy range considered to be between 4:1 and 1:1 [52].

For comparison, our study rated the EI of athletes with the daily energy expenditure, basal metabolic rate, energy expenditure during training exercises, and the daily energy expenditure of all study participants. The basal metabolic rate was calculated applying the Harris and Benedict equations [53]. The levels of physical activity were assessed according to the recommendations along with the evaluation algorithm established by the American College of Sports Medicine and the American Dietetic Association, Dietitians of Canada [54]. The benchmark data of the basic physical exertion and the activity codes, coupled with the metabolic equivalents (in kcal/kg/h) for specific physical activities, were applied in the observational study. These proceedings were supplemented by the research of Ainsworth et al. [55], and the data were handled depending on the specific activity. Thus, the physical activity level was calculated as the ratio between the daily energy expenditure and the basal metabolic rate, as mentioned by FAO/WHO/UNU [56]. Eventually, in order to calculate the total energy requirement, the basal metabolic rate was then multiplied by the suitable physical activity factor.

The measurements of BW (in kg) and standing height (achieving ±1 cm accuracy) were assessed using the bioelectrical impedance analysis (BIA) via the body composition analyzer X-Scan Plus (the International Organization for Standardization adopted by the European Union (EN-ISO): 13488 [57], Seoul, Republic of Korea).

#### 2.2.2. Blood Collection

During the periodical medical examination, blood samples were collected from the selected athletes by a trained phlebotomist at an accredited LSC clinical laboratory (medical laboratory license number 2545; Vilnius, Lithuania). In order to control the possible effect of exercise on the hematological results, study participants were asked to avoid physical effort for 24 h before phlebotomy, as well as to be hydrated and fasted.

In laboratory settings, the hematology profile of athletes (red blood cell (RBC) count), red blood cell distribution width (RDW), hemoglobin (Hb), mean corpuscular hemoglobin concentration (MCH), hematocrit (Ht), white blood cell (WBC) count, lymphocytes (Lym), monocytes (Mo), platelet (PTL) count, mean platelet volume (MPV), and plateletcrit (PCT)) was assessed via the Nihon Khoden automated hematology analyzer (MEK6400-K, Tokyo, Japan). Also, for the study data analysis, an additional platelet function-related variable, namely, the platelet-to-hemoglobin ratio (PHR), was calculated by dividing the platelet count by the hemoglobin concentration [58,59].

The blood samples of study participants were tested individually, with the possibility of repeating and verifying the unusual results. The laboratory monitored the precision and accuracy of all blood samples analyzed.

### 2.3. Statistical Data Analysis

The statistical examination was completed utilizing the Statistical Package for the Social Sciences (IBM SPSS Statistics) version 25.0 for Windows (IBM Corp, Armonk, NY, USA).

Firstly, the Kolmogorov–Smirnov test as an a priori statistical approach was used to test for data normality. In the second place, the primary empirical data obtained from this study were classified according to both bivariate and multivariate analyses.

For the bivariate analysis, the means ± standard deviations (SDs) for normally distributed data were calculated, and a *t*-test, along with Cohen’s D (*d*) effect sizes, including the Pearson’s correlation coefficient (r), was used for data analysis. More specifically, nutritional adequacy was verified by contrasting macronutrient intakes to the RDAs. A *t*-test coupled with *d* estimates [60] (‘trivial effect’: 0 ≤ *d* < 0.2; ‘small effect’: 0.2 ≤ *d* < 0.5; ‘moderate effect’: 0.5 ≤ *d* < 0.8; ‘large effect’: 0.8 ≤ *d*) as effect sizes were applied to resolve whether there was a significant difference between the means of the two subgroups (e.g., EI and the essential macronutrient intake amounts vs. the daily energy expenditure and RDAs for macronutrients). Also, the *t*-test was used to determine whether the athletes competing in different sports (aerobic vs. anaerobic) had different nutritional and hematological profiles. In addition, in order to assess the correlation between the PTL count (×10^9^/L) and the MPV (fL) and Hb (g/dL), the r values were calculated and interpreted as follows: ‘negligible correlation’ (0 ≤ r < 0.3); ‘low to moderate positive correlation’ (0.3 ≤ r < 0.7); ‘high to very high positive correlation’ (0.7 ≤ r ≥ 0.9).

For multivariate analysis, our study carried out multiple logistic regression analyses with the binary dependent variables around four groups covering the indices of blood profiles, namely, ‘244 × 10^9^/L ≤ PTL count > 244 × 10^9^/L’, ‘0.19% ≤ PCT > 0.19%’, ‘14.7 g/dL ≤ Hb > 14.7 g/dL’, ‘16.4 ≤ PHR > 16.4’. The independent variables, namely, ‘3307 kcal/day ≤ EI > 3307 kcal/day’, ‘395.4 g/day ≤ carbohydrates > 395.4 g/day’, ‘115.6 g/day ≤ protein > 115.6 g/day’, ‘143.9 g/day ≤ total fat > 143.9 g/day’, ‘50.3 g/day ≤ saturated FA > 50.3 g/day’, ‘720 mg/day ≤ cholesterol > 720 mg/day’, ‘1.04 g/day ≤ omega-3 FA > 1.04 g/day’, ‘19.9 g/day ≤ omega-6 FA > 19.9 g/day’ were adjusted to the dichotomous form. Thus, the units of measurement of dependent and independent variables were modified to the dichotomous form based on cut-off values that were set by referring to the median values of the group of athletes studied. All logistic regression models were adjusted for the sex and sports discipline of professional athletes.

In all the statistical tests applied to data analysis, a two-tailed *p*-value ≤ 0.05 was considered statistically significant. The SPSS software, along with the free and open-source software LibreOffice version 7.6.4, was used for the visualization of statistical data.

## 3. Results

### 3.1. Characteristics of Athletes

Out of 82 high-performance athletes of a mean age of 19.8 ± 2.2 years who constituted the representative sample, 25.6% were females and 74.4% were males. From a sporting perspective, study participants were divided into aerobic and anaerobic athletes at 58.5% and 41.5%, respectively. The range for training practice equaled 6.8 ± 2.5 years, the number of training sessions was 6 days per week, and the duration of daily workout was 173.2 ± 66.3 min on average. The mean standing height and body weight of the athletes involved in the study were 181 ± 13 cm and 71.6 ± 15.4 kg, respectively. The extent of the workload of the athletes was in agreement with the validated training plans. For the purpose of assessing the workload plans of the athletes studied, account has been taken of the training plans for athletes that were officially confirmed by the LTOK. More detailed data referring to the exercise plans designed for high-performance athletes is provided in Table 1.

### 3.2. Nutritional Status

Table 2 presents the reported usual mean intakes of energy, carbohydrates, protein, fats, saturated FAs, cholesterol, monounsaturated FAs, and polyunsaturated FAs in high-performance athletes.

For elite athletes, the recorded mean consumption levels of energy, carbohydrates, protein, and fat were 49 ± 15 kcal/kg/day, 5.4 ± 1.9 g/kg/day, 1.6 ± 0.5 g/kg/day, and 40.3 ± 6.7% of energy intake, respectively. Overall, the comparison of the results with RDAs showed that the levels of the basic macronutrients consumed by athletes were not balanced, i.e., carbohydrate intake was too low (*d* = −1.63) while fat consumption was excessive (*d* = 1.06). For anaerobic athletes, the intakes of saturated FA and monounsaturated FA and dietary cholesterol were expressed at higher levels compared to those in the athletes competing in endurance sports (0.001 ≤ *p* ≤ 0.05). Specifically, in aerobic and anaerobic athletes, the mean daily cholesterol intake ranged from 698 ± 391 mg to 982 ± 478 mg, respectively. The dietary cholesterol intake of this magnitude, usually originating from a typical meal high in saturated fat, exceeded the previously existing RDA [61] for cholesterol (300 mg/day) by 2–3 times.

On the contrary, in terms of effect sizes (*d*), a severe deficiency of polyunsaturated FA and omega-3 FA was observed in athletes’ nutrition (*d* = −1.86 and *d* = −8.50). In addition, when the optimum ratio of omega-6 FA and omega-3 FA should be 4:1, this study identified an excess ratio (19.1:1) of almost five times the recommended one, regardless of the sport being cultivated by athletes.

### 3.3. Blood Analysis

Table 3 shows the analysis of the blood samples of both aerobic and anaerobic athletes studied. Essential hemogram parameters (RBC count, Hb, MCV, Ht, MCH, WBC count, Lym, Mo, PTL count, PCT, MPV) in athletes were within the normal range. Taking into account the variations found in the trivial effect size (*d*) from −0.2 to 0.19, it can be confirmed that the baseline blood parameters fluctuated within the standards, especially when compared to the recommended lower bound (2.5th percentile) and upper bound (97.5th percentile) of the RIs and their 90% CIs established for the athletes’ hematological parameters [62]. On the other hand, the analysis of blood samples revealed that red cell distribution width (RDW 11.6 ± 0.7%) was below the minimum recommended threshold (RDW 12.1–14.3) for athletes (*d* = −2.3). Thus, when this score exceeded the recommended range, there was a possibility that study participants had a nutrient deficiency.

**Figure 2:**
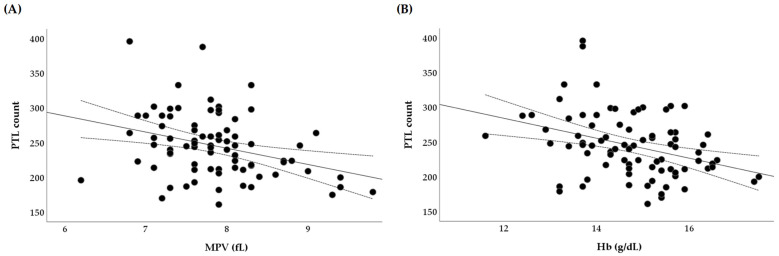
(**A**,**B**) The correlation between the platelet (PTL) count (×10^9^/L) and the mean platelet volume (MPV) (fL), and hemoglobin (Hb) (g/dL).

The results of this study showed a reverse correlation between the PTL count and the MPV (r = −0.3, *p* = 0.003) (Figure 2A). It should be highlighted that the mean platelet volume (MPV) is a measurement of the mean platelet size. Although it is an indicator of platelet activation, a significant inverse correlation between the MPV and PTL count can be found [63]. In healthy athletes and under certain conditions, lower platelet counts may be related to higher MPV, and vice versa, taking into account the fact that the medulla ossium responds to a greater demand by releasing younger and larger thrombocytes. In other words, lower levels of platelet count and higher levels of MPV may serve as signs of the athletes moving to a better recovery and regeneration between the intense physical loads [64].

In addition, our study explored the platelet-to-hemoglobin ratio (PHR = 16.8 ± 3.9) as a novel indicator for stress and inflammation [58,59]. In this case, higher PHR may imply increased PTL counts and/or decreased Hb levels, to which the athlete’s body may respond with increased inflammation induced by physical exertion or chronic stress. Thus, the results obtained from our study have confirmed the inverse correlation between PTL and Hb (r = −0.4, *p* = 0.001) (Figure 2B). This study, in its subsequent stages, assessed a possible relationship between the nutritional status and the indices of the athletes’ blood profiles, including the platelet-to-hemoglobin ratio.

### 3.4. Associations Between Dietary Intakes and Platelet Variables

**Figure 3:**
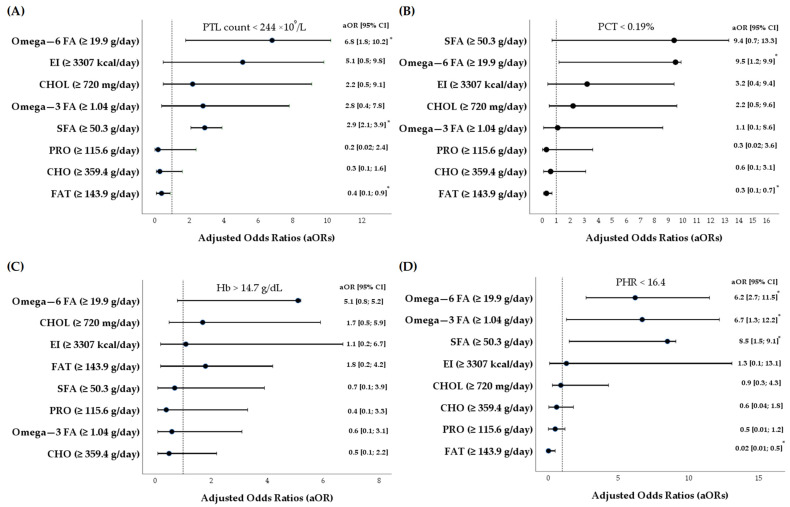
(**A**–**D**) A graphic representation of multivariate logistic regression models (dependent variables: PTL count (×10^9^/L), PCT (%), Hb (g/dL), and PHR; independent variables: EI (kcal/day), CHO (g/day), PRO (g/day), FAT (g/day), SFA (g/day), CHOL (mg/day), omega-6 FA (g/day), and omega-3 FA (g/day). A dotted line represents the adjusted odds ratio (AOR) equal to 1. If AOR < 1 and AOR ≠ 1, it means that there is an inverse association between the independent and dependent variables. If AOR > 1 and AOR ≠ 1, it means that there is a positive association between the independent and dependent variables. The multivariate logistic regression models (**A**–**D**) were adjusted for athletes’ sex and sports discipline. *—*p* ≤ 0.05, 95% CI—95% confidence interval, CHO—carbohydrates, CHOL—cholesterol, EI—energy intake, FAT—fats, PCT—plateletcrit; PHR—the platelet-to-hemoglobin ratio; PTL—platelet, PRO—protein, FA—fatty acids, SFA—saturated FA.

As shown in Figure 3A–D, further multivariate logistic regression analyses revealed significant relationships between the dietary intakes of polyunsaturated FA and the indices of blood profiles in a cohort of elite athletes. Following the adjustment of logistic regression models for athletes’ sex and sports discipline [65,66,67], in relation to slightly higher levels of blood PTL counts (>244 × 10^9^/L), the AOR for higher intake of omega-6 FA among high-performance athletes was 6.8 (95% CI 1.8; 10.2, *p* = 0.016). Similarly, an association was also found between PCT (>0.19%) and omega-6 FA intakes (AOR 9.5, 95% CI 1.2; 9.9, *p* = 0.029).

Finally, the PHR (<16.4), as a blood-based biomarker calculated by dividing PTL counts by Hb levels and used for an indirect assessment of systemic stress, immune activity, and inflammation [58,59], was inversely associated with higher intake levels of dietary omega-3 FA (AOR 6.7, 95% CI 1.3; 12.2, *p* = 0.029), omega-6 FA (AOR 6.2, 95% CI 2.7; 11.5, *p* = 0.009), and saturated FA (AOR 8.5, 95% CI 1.5; 9.1, *p* = 0.020) (Figure 3D).

## 4. Discussion

The aim of this study was to assess the levels of macronutrients consumed by high-performance athletes and to associate the latter with the basic indicators of a hemogram. The study found that macronutrients, namely, protein and carbohydrates, consumed by athletes were not associated with the platelet count, plateletcrit, or platelet-to-hemoglobin ratio. Nevertheless, within the segment of elite athletes under analysis, our study observed the imbalances between the intake of basic macronutrients, manifested by the deficit consumption of carbohydrates (5.3–5.7 g/kg of body weight) and an excessive consumption of the total dietary fat (38.5–42.8% of EI), exclusively saturated FA (13.4–14.3% of EI), and dietary cholesterol (698–982 mg/day). A typical isocaloric Western-type diet consumed by athletes contained a higher percentage of calories from omega-6 FA (around 5.4–5.9% of EI) and a lower percentage from omega-3 FA (around 0.3% of EI). Given that the ideal human omega-6 to omega-3 FA ratio is commonly deemed to be between 1:1 and 4:1, an athlete’s ‘Western diet’ was heavily skewed, with a ratio fluctuating from 18.9:1 to 19:4 in favor of omega-6 FA.

Our study revealed a positive association between the omega-3 FA intake and the platelet-to-hemoglobin ratio, which has been indirectly related to the athletes’ poor nutrition status, inflammation, and oxidative stress. On the other hand, although this study did not reveal a positive relationship between the omega-3 FA consumption and the total platelet count or plateletcrit, our findings were partly consistent with the data collected from the scientific research, with scientists speculating that FA obtained from a healthy or balanced diet can trigger or inhibit platelet aggregation. Thus, omega-3 FA, mainly found in fish oil and walnuts, such as eicosatetraenoic acid (EPA) and docosahexaenoic acid (DHA), have been demonstrated to boost the antiplatelet function and the immunity [68,69,70,71,72,73]. More specifically, these useful FAs can integrate into the membranes of platelet cells and subsequently modulate their fluidity and activation [38]. This antiplatelet effect of omega-3 FA can be explained by the fact that EPA and DHA can change the membrane fluidity, the expression of a regulating receptor, and the production of thrombin [74]. Resolvins (resolvins E and D), as endogenous lipid mediators derived from omega-3 FAs, are capable of decreasing and inhibiting thromboxane-induced thrombocyte aggregation. Although the antiplatelet effect of oils or marine fish has been confirmed in healthy study participants, the dose used was considered to be a determining factor in serving a benefit, especially given that the low and high doses do not always indicate a benefit [37,75,76,77,78,79,80,81,82].

Also, research-based evidence has revealed that the increase in the omega-6 to omega-3 FA ratio can shift the balance into a proaggregatory state [83]. This study revealed similar findings in relation to the excessive ratio of omega-6 to omega-3 FA resulting from higher consumption of omega-6 FA among high-performance athletes. In addition, when higher platelet counts are linked to an increased risk of clotting, our study found an association between the higher intake of omega-6 FA and the total platelet count as well as plateletcrit. Thus, higher consumption of omega-6 FA may serve as the potential precursor in blood coagulation and vasoconstriction. In this context, unconstrained or disproportionate blood clotting is not appropriate for athletes, as it may elevate the risk of serious blood clots (thromboembolism) depending on conditions, namely, muscle damage, dehydration, psychological stress, or immobility during travel, regardless of the healthy nature of athletes.

Furthermore, this study showed that lower levels of the platelet-to-hemoglobin ratio were related to an increased consumption of omega-3 FA, omega-6 FA, and saturated FA in high-performance athletes under our analysis. Preliminary, these findings refer to the possible fact that the activity of the immune system is not impaired by the chemical nature of the FA consumed by elite athletes. In agreement with these findings, it should be noted that as the predecessor to pro-inflammatory eicosanoids, high levels of omega-6 FA intake cannot directly cause inflammation, mostly in healthy people; however, high omega-6 FA intake itself may restrict the anti-inflammatory outcomes from the omega-3 FA. Thus, in the context of inflammation, the relationship between omega-3 FA and omega-6 FA and their lipid intermediaries remains mixed and still misunderstood, especially in athlete populations.

Additionally, the results originating from our study did not match the authors’ reports that food rations high in saturated fats may amplify the platelet function, bringing about the pro-thrombotic conditions [84]. These contradictory results can be explained by the disparities observed in populations of non-sporting people and professional athletes. More specifically, in terms of the non-physically active population, a high-fat diet may increase the levels of ‘bad’ low-density lipoprotein (LDL) cholesterol, resulting in hyperlipidemia, which, in turn, can promote platelet activity. Secondly, the increased platelet activation may take place via the occurrence of changes in platelet phospholipid content and the activation of signaling pathways similar to P2Y12 [85,86]. In this case, it should be highlighted that almost all of the athletes involved in our study reported high consumption levels of fat and saturated fatty acids. Other researchers have revealed the same findings [87]. However, to date, there has been no scientific evidence that a high-fat diet leads to overweight athletes [88]. On the contrary, there is evidence that the use of a high-fat diet can increase the levels of circulating homocysteine, which serves as a potential risk factor for the development of cardiometabolic disorders [89]. The athletes’ demand to reduce the consumption of saturated FA may also be confirmed by the results obtained from other empirical studies, which refer to the fact that high intakes of saturated FA in athletes were related to greater muscle soreness and impaired recovery by triggering adipose tissue inflammation via receptors like TLR4 [90].

Therefore, the athletes may reduce this potential inflammation by using polyunsaturated FA such as omega-3 FA. Also, reducing the consumption of foods rich in saturated FA and increasing the consumption of whole grains, low-fat dairy products, lean poultry and meats, fish, fruits, and vegetables could bring more nutritional benefits to athletes in order to enhance the adaptation to intense physical loads [28,90]. Within this framework, the cohorts of athletes become distinctive in line with the International Society of Sports Nutrition (ISSN) position stand, establishing the following: (1) ‘Athletes may be at a higher risk for omega-3 FA insufficiency’; (2) ‘Diets rich in omega-3 FA, including supplements, are effective strategies for increasing omega-3 FA levels’; (3) ‘Also, omega-3 FA supplementation can positively affect various immune cell responses in athletic populations’ [73].

This study had several limitations. In particular, minor biases could have occurred due to the methodology used in the dietary assessment process, on the basis of which the accuracy of the final dataset could have been affected by specific factors such as the training status, nutrition-related beliefs, the level of competition, or memory gaps in the composition of the diet consumed or recognizing food brands. Secondly, the search for associations between independent variables and platelet variables may have been partially distorted by potential confounders related to protein consumption. More precisely, some research has found that dietary proteins, particularly those rich in certain amino acids like glycine and arginine, and platelet function were interconnected [71,72]. Thirdly, the relatively moderate, although representative, sample size of our study can restrict the extrapolation of the results of this study to a less physically active segment of the human population. Fourthly, given that this exploratory study used a cross-sectional analysis, the results obtained from this study cannot refer to causal relationships between the variables analyzed. In this case, taking into account our novel study findings, undertaking further longitudinal and/or experimental studies in design could provide additional pronounced scientific robustness. Also, the additional measurement of biomarkers (i.e., the omega-3 index on erythrocytes and lipid profiles on the plasma) could make the correlations with the hematological indicators more precise.

## 5. Conclusions

Considering that increased physical loads, even within the limits of physiological norms, may have an adverse impact on both the total platelet count and their functional activity, the results of this study highlighted important associations between the dietary fat profile and the platelet-related variables observed in a sample of high-performance athletes.

Following the adjustment to athletes’ sex and sports discipline, the outcomes related to slightly higher levels of blood platelet counts and plateletcrit were associated with higher intakes of omega-6 fatty acids. Moreover, a higher platelet-to-hemoglobin ratio, as a novel indirect blood-based biomarker pronouncing potential inflammatory processes in the body, had a reverse relationship with higher intake levels of dietary omega-3, omega-6, and saturated fatty acids among elite athletes.

In this context, whilst high-performance athletes consume high levels of saturated fatty acids, including dietary cholesterol, and low omega-3 fatty acids during their training process, this dietary behavior related to the intake of different dietary fatty acid fractions still maintains unrecognized metabolic pathways linked to the possible positive benefits or negative outcomes for the immune system, especially in the event of progressive high physical workloads among elite athletes. Thus, the findings originating from this study may benefit the athletes exposed to extreme physical exposure, coaches, and healthcare providers, or initiate further research into health-related consequences of the intakes of both polyunsaturated fatty acids and saturated fatty acids, including dietary cholesterol.

Finally, a holistic understanding of the platelet function, which exclusively includes the role of nutrition, can enhance the perception of thrombosis and shed light on the development of new intervention or treatment strategies. The prospect of personalized nutrition targeted at the professional athletes’ segment may provide an innovative opportunity to increase their capacity to manage platelet function via diet, while stressing the importance of further empirical experimental research in this dynamic and vital biomedical field, too.

## Figures and Tables

**Table 1:** The exercise plans for high-performance athletes.

Sports	Skiing and Biathlon	Boxing, Graeco-Roman Wrestling and Judo	Road Cycling	Swimming (50–400 m) and Canoe Paddling (500–2000 m)	Modern Pentathlon	Basketball
Performance investigation by month	August	May–June	April	March–May	June	May
Macrocycle phase	Preparation	Preparation	Preparation	Preparation	Preparation	Preparation
Stage	Basic training	Special training	Special training	Special training	Special training	Special training
Days of exercise per month	27	27	26	25–27	26	27
Total physical training (hours per month)	31–32	36–41	36	40–41	40	41
Special training is categorized into five zones of intensity depending on ATP production in the muscles						
Zone 1: Aerobic strength endurance training, recovery (pulse rate − 130 ± 10 bpm, lactate level up to 2 mmol/L)	26–36%	12–18%	18%	18–20%	20%	18%
Zone 2: Aerobic strength training (pulse rate − 150 ± 10 bpm, lactate level is 2–4 mmol/L), muscular power increase at the anaerobic threshold	37–48%	26–30%	50%	40–50%	50%	42%
Zone 3: Mixed aerobic and anaerobic glycolytic strength training (pulse rate −170 ± 10 bpm, lactate level is 4–12 mmol/L), increase of VO_2_max	15–17%	40–50%	32%	29–35%	25%	32%
Zone 4: Anaerobic glycolytic strength training (pulse rate ≥181 bpm, lactate level up to 21 mmol/L)	6–10%	8–10%	–	5%	3%	5%
Zone 5: Anaerobic phosphocreatine strength training (lactate level is 1.5–6 mmol/L)	2–3%	2–4%	–	1–2%	2%	3%

The exercise time was assigned for intensity zones (%); ATP—adenosine triphosphate.

**Table 2:** Macronutrient intakes in high-performance athletes according to sport branches.

Energy Intake and Macronutrients	Endurance Athletes (*n* = 48)	Strength-Power Athletes (*n* = 34)	RDAs	*d*
Energy intake (kcal/kg/day)	49 ± 15	50 ± 16	50 ± 8 [49; 53]	−0.07
Carbohydrates (g/kg/day)	5.5 ± 1.9	5.3 ± 1.9	7–10	−1.63
Carbohydrates (% of EI)	47.3 ± 7.4 **	43.2 ± 6.2	45–65	−1.31
Protein (g/kg/day)	1.6 ± 0.5	1.7 ± 0.5	1.2–2.0	−0.02
Protein (% of EI)	14.2 ± 3.1	14.0 ± 2.6	12–20	−0.82
Lipids (g/kg/day)	2.0 ± 0.8	2.3 ± 0.8	1.0–1.5	1.06
Lipids (% of EI)	38.5 ± 6.9	42.8 ± 5.7 **	25–35	1.53
Saturated FA (g/day)	45.5 ± 19.7	60.4 ± 20.5 ***	—	—
Saturated FA (% of EI)	13.4 ± 3.4	14.3 ± 2.5	≤10	1.22
Cholesterol (mg)	698 ± 391	982 ± 478 ***	—	—
Monounsaturated FA (g/day)	63.5 ± 23.6	97.2 ± 47.6 ***	—	—
Polyunsaturated FA (g/day)	20.1 ± 10.7	27.2 ± 12.0 **	—	—
Polyunsaturated FA (% of EI)	5.9 ± 2.4	6.3 ± 1.7	6–10	−1.86
Omega-6 FA (g/day)	18.4 ± 9.8	25.4 ± 11.5 **	—	—
Omega-6 FA (% of EI)	5.4 ± 2.2	5.9 ± 1.7	5–8	−0.19
Omega-3 FA (g/day)	1.1 ± 0.8	1.3 ± 0.5	—	—
Omega-3 FA (% of EI)	0.3 ± 0.2	0.3 ± 0.1	≥2	−8.50
Omega-6 FA/Omega-3 FA ratio	18.9 ± 8.1	19.4 ± 6.1	1:1–4:1	2.07

Data are presented as means ± standard deviations (SDs). The measure of Cohen’s D displays the effect size (*d*) of the mean differences in dietary nutrient intakes after equating them to the RDAs. FA—fatty acids, RDA—recommended dietary allowance. All *p*-values (*p*) obtained from the *t*-test reporting the significance of differences between the dietary nutrient intakes depending on the sport branches. **—*p* ≤ 0.01, ***—*p* ≤ 0.001.

**Table 3:** Hemogram of elite athletes.

Parameter	Endurance Athletes (*n* = 48)	Strength-Power Athletes (*n* = 34)	RIs for Physically Active Individuals 2.5th–97.5th Percentile	*d*
RBC count (×10^12^/L)	5.1 ± 0.4	5.0 ± 0.5	4.4–5.6	0.14
Hb (g/dL)	14.7 ± 1.1	14.9 ± 1.2	13.0–16.1	0.17
MCV (fL)	86.4 ± 3.4	87.2 ± 3.8	80.9–94.9	−0.19
Ht (%)	44.4 ± 3.7	44.0 ± 3.7	39.5–48	0.13
MCH (pg)	28.6 ± 1.5	29.4 ± 1.6	26.1–31.6	0.01
RDW (%)	11.7 ± 0.8	11.6 ± 0.7	12.1–14.3	−2.3
WBC count (×10^9^/L)	6.9 ± 1.6	7.2 ± 1.9	4.5–10.1	−0.17
Lym (%)	34.0 ± 6.3	34.5 ± 8.4	20–44	0.19
Mo (%)	6.9 ± 1.8	6.6 ± 1.6	2.0–9.5	0.11
PTL count (×10^9^/L)	247 ± 49	241 ± 42	140–337	0.10
PCT (%)	0.19 ± 0.03	0.20 ± 0.03	0.12–0.41	−0.20
MPV (fL)	7.8 ± 0.6	7.9 ± 0.7	5.9–9.9	0.14

Data are presented as means ± standard deviations (SDs). Hb—hemoglobin; Ht—hematocrit; Lym—lymphocytes; MCH—the mean corpuscular hemoglobin concentration; Mo—monocytes; MPV—the mean platelet volume; PCT—plateletcrit; PTL—platelet; RBC—red blood cells; RIs—reference intervals [62]; RDW—the red blood cell distribution width; WBC—white blood cells; *d*—the effect size.

## Data Availability

The data presented in this study are available on request from the corresponding author due to sharing them would go against ethical considerations, such as study participants’ privacy and consent.

## Abbreviations

ATP	Adenosine triphosphate
BIA	Bioelectrical impedance analysis
BW	Body weight
CI	Confidence interval
CVD	Cardiovascular disease
DHA	Docosahexaenoic acid
EI	Energy intake
EN	European Union
EPA	Eicosapentaenoic acid
FA	Fatty acids
Hb	Hemoglobin
Ht	Hematocrit
ISO	International Organization for Standardization
ISSN	International Society of Sports Nutrition
LB	Lower bound
LDL	Low-density lipoprotein
Lym	Lymphocytes
LSC	Lithuanian Sports Centre (LSC)
LTOK	Lithuanian National Olympic Committee
MCH	Mean corpuscular hemoglobin concentration
Mo	Monocytes
MPV	Mean platelet volume
NO	Nitric oxide
OR	Odds ratio
PCT	Plateletcrit
PHR	Platelet-to-hemoglobin ratio
PTL	Platelet
RBC	Red blood cells
RDA	Recommended dietary allowance
RDW	Red blood cell distribution width
SD	Standard deviation
SPSS	Statistical Package for the Social Sciences
TxB3	Thromboxane B3
UB	Upper bound
WBC	White blood cells
